# Associations between antipsychotics and risk of violent crimes and suicidal behaviour in personality disorder

**DOI:** 10.1136/ebmental-2022-300493

**Published:** 2022-10-25

**Authors:** Kimmo Herttua, Mike Crawford, Tapio Paljarvi, Seena Fazel

**Affiliations:** 1 Public Health, University of Southern Denmark - Campus Esbjerg, Esbjerg, Denmark; 2 Department of Brain Sciences, Imperial College London Faculty of Medicine, London, UK; 3 Psychiatry, Oxford University, Oxford, UK; 4 Psychiatry, Niuvanniemi Hospital, Kuopio, Finland; 5 Psychiatry, University of Oxford, Oxford, UK

**Keywords:** Personality disorders, Adult psychiatry, Suicide & self-harm, Forensic psychiatry

## Abstract

**Background:**

Despite uncertain benefits, people with personality disorder are commonly treated with antipsychotic medication.

**Objective:**

To investigate the association between antipsychotics and violent crimes and suicidal behaviour in individuals with personality disorder.

**Methods:**

We used nationwide Danish registries to identify all individuals with diagnosed personality disorder aged 18–64 years during 2007 to 2016. Antipsychotics were recorded in dispensed prescriptions, and individuals were followed up for police-recorded suspicions for violent crimes and healthcare presentations of suicidal behaviour. We applied a within-individual design where outcome rates for individuals with personality disorder during medicated periods were compared with rates during non-medicated periods.

**Findings:**

The cohort included 166 328 people with diagnosed personality disorder, of whom 79 253 were prescribed antipsychotics, presented at least one outcome and were thus included in the within-individual analyses. Compared with periods when individuals were not on antipsychotic medication, violent crime suspicions were 40% lower (incident rate ratio (IRR) 0.60, 95% CI 0.55 to 0.63) in men and 10% lower (IRR 0.90, 95% CI 0.79 to 1.01) in women, while rates of suicidal behaviour were 32% lower both in men (IRR 0.68, 95% CI 0.66 to 0.71) and in women (IRR 0.68, 95% CI 0.65 to 0.70). In subgroup analyses, the magnitude of the association varied across specific personality disorders for criminal outcomes but less for suicidal behaviour, with largest association in dissocial personality disorder for violent criminality (IRR 0.53, 95% CI 0.47 to 0.59).

**Conclusions:**

Treatment with antipsychotics was associated with reduced risks for violent crime suspicions and suicidal behaviour among individuals with personality disorder.

**Clinical implications:**

Potential effects of antipsychotics on suicidal behaviour and violence should be taken into account when considering treatment options for people with personality disorders.

**WHAT IS ALREADY KNOWN ON THIS TOPIC**

- Associations between antipsychotics and adverse outcomes have been reported in other populations, including in schizophrenia-spectrum disorders and bipolar disorder, but not in people with personality disorder.

**WHAT THIS STUDY ADDS**

- The findings of this large population-based cohort study show that, compared with periods when individuals were not on antipsychotic medication, rates of violent criminality and suicidal behaviour were substantially lower when dispensed antipsychotics.
- In subgroup analyses, the magnitude of the association varied across specific personality disorders for criminality but less for suicidal behaviour, with larger effects in dissocial personality disorder.

**HOW THIS STUDY MIGHT AFFECT RESEARCH, PRACTICE OR POLICY**

- Antipsychotics could be considered for prevention of suicidal behaviour and violence sin people with personality disorders if triangulated with other evidence.

## Introduction

In the adult general population in Western countries, it is estimated that around one in ten persons have personality disorders.^1^ People with personality disorders have higher morbidity, mortality and rates of other adverse outcomes, including violence perpetration, than general population comparisons^2 3^ with high societal costs.^2 4^

In particular, there is replicated evidence that personality disorder is associated with higher risk for criminal^5 6^ and suicidal behaviours.^7 8^ Reviews have reported a threefold increase in the odds of violent outcomes in individuals with all personality disorders compared with general population controls, with substantially higher risks in antisocial personality disorder.^5^ In addition, rates of self-harm and suicide mortality, compared with community controls are fourfold higher.^7 9^

The treatment of personality disorder has focused on borderline personality disorder,^10^ which is the most common subcategory in women and a common presentation to emergency care for self-harm and psychiatric hospitalisation. Psychological and psychosocial treatments are primarily recommended.^10 11^ There is an absence of clear evidence suggesting benefits for any class of psychotropic medication.^12 13^ Despite this, two-thirds of the patients with borderline personality diagnosis are reported to be prescribed long-term antipsychotic medication.^14 15^ At the same time, the evidence base for psychological treatments is not strong, and concerns have been raised about risk of bias, publication bias, and limited information about long-term effectiveness.^16^

In addition, treatment studies have concentrated on symptomatic improvement and some have investigated functional outcomes, such as quality of life.^11^ However, they have not examined longer-term real-world outcomes with important consequences for individuals and public health. Among these, criminal or suicidal behaviour are important due to disruption of healthcare, chronic morbidity due to injury and wider impact on carers and families, and victims of crime. However, these outcomes have been studied in other populations using pharmacoepidemiological designs. In particular, associations between antipsychotics and crime outcomes have been reported in general,^17 18^ clinical^19^ and high-risk populations^20^ using population registers. For suicidal outcomes, effects are less clear as sample sizes have been small.^21^ These studies have accounted, to some extent, for confounding by indication using within-individual designs, where medication periods are compared with non-medication periods in the same person.

## Objective

Therefore, using high quality Danish population registers, we aimed to investigate whether antipsychotics are associated with risk of violent crime and suicidal behaviour in individuals with a diagnosed personality disorder using a within-individual design.

## Methods

### Study design and participants

All data were obtained by the linkage of Danish national registers. The Danish Civil Registration System was established in 1968 and comprises information on all live-born children and new residents in Denmark, who are assigned a Civil Personal Register (CPR) number. The CPR number is used to register usage of healthcare services, and it enables Statistics Denmark to carry out linkages between various data sources at individual level. All registers have full national coverage, and information from the registers are anonymised when used for research. The use of anonymised national registers for research does not require consent from participants according to Danish legislation. The authors assert that all procedures contributing to this work comply with the ethical standards of the relevant national and institutional committees on human experimentation and with the Helsinki Declaration of 1975, as revised in 2008. All procedures involving human subjects/patients were approved by the Danish Data Protection Agency and the Danish Health Data Authority (18/16328).

In this study, the participants were restricted to individuals between 18 and 64 years of age during the follow-up period 2007–2016. The follow-up started from 1 January 2007 or from 1 January after the point of turning 18 years of age. Participants became censored when reaching the age of 65, moving abroad, at death or at the end of the study period. From this full nationwide population sample, we then identified individuals who had been diagnosed with personality disorder (ICD codes F60.0–F60.9 and F61) using the International Statistical Classification of Diseases and Related Health Problems, Tenth Revision (ICD-10) through the Danish Psychiatric Central Research Register which contain information on all inpatients and outpatients for psychiatric hospitals and outpatient services in Denmark.

### Medications

We extracted data about treatment with antipsychotics, identified in the Danish National Prescription Registry according to the Anatomical Therapeutic Chemical (ATC) classification system. These data include all dispensed medication (ie, those medicines prescribed and collected). Antipsychotics were defined as drugs with ATC codes N05A. Out of 79 253 individuals, who had at least one prescription during the study period, 44 156 (55%) had dispensed quetiapine (ATC code N05AH04), 20 129 (25%) olanzapine (ATC code N05AH03), 17 598 (22%) risperidone (ATC code N05A×08), 3593 (4%) clozapine (ATC code N05AH02) and 53 315 (66%) other antipsychotics. In additional analyses, we also included prescriptions of lithium (ATC code N05AAN01) which had been dispensed for 7739 (10%) individuals. For the analysis adjustments, we also extracted information on treatment with antidepressants (ATC code N06A) and hypnotics and anxiolytics (ATC codes N05B and N05C). Furthermore, for the negative control analysis, we extracted data about treatment with adrenergic inhalants (R03A), a commonly used medication class with negligible psychotropic effects.

### Outcomes

The primary outcomes were violent crime suspicions (also described as violent crimes/criminality in this paper) and suicidal behaviour. Data on suspected crimes were extracted from the Administrative System of the National Police including data on all crimes with the dates of the suspected crimes reported to police, also including those not pursued after being reported. A suspected crime follows an initial investigation where a decision is made to pursue a charge. The outcome of suspected crimes instead of convictions is more sensitive as a proportion of such suspicions are dropped on the basis of offenders’ mental health concerns.

Data on suicidal behaviour were obtained from the Danish National Patient Register, the Danish Psychiatric Central Research Register and the Cause of Death Register. Suicidal behaviour refers to completed suicides and suicide attempts. Suicide deaths were denoted by suicide recorded as the underlying cause of death on the death certificate. We identified suicide attempts using a validated algorithm developed using the same registers which is based on specific codes of ICD-10 of primary or secondary inpatient and outpatient discharge diagnoses at somatic and psychiatric hospitals indicating suicide attempt, intoxication and injury and self-harm.^22 23^ The following contacts with somatic and psychiatric hospitals were recorded as suicide attempts: (1) all hospital contacts with main ICD-10 diagnosis with codes X60-X84 (intentional self-poisoning and intentional self-harm), (2) all hospital contacts with reason for contact as NOMESCO (Nordic Medico-Statistical Committee) Code 4 (suicide attempt or self-harm), (3) all hospital contacts with a main diagnosis in chapter F60.0–F60.9 or F61 (personality disorders) and with concomitant diagnosis of intoxication with codes T36–T50 (all drugs and biological substances, independent of kind of intoxication) and T52–T60 (damaging effect of non-medical substances, excluding alcohol and food poisoning) and (4) all hospital contacts with a main diagnosis in chapter F60.0–F60.9 or F61 (personality disorders) and with concomitant diagnosis of cutting of the lower arms, wrist and hands with codes S51, S55, S59, S61, S65 and S69.

### Statistical analyses

We initially fitted a series of longitudinal Poisson regression models to the full sample of individuals who had been diagnosed with personality disorder during the 10 years study period (n=166 328). These models captured between-individual associations: comparisons of the outcome rates between individuals who had been on antipsychotic medication at some point during the study period versus those who were not on medication. In additional analyses, we also included individuals who had been dispensed solely prescriptions of lithium. The between-individual analyses were adjusted for use of antidepressant and hypnotics/anxiolytics, age as a continuous variable, living arrangement and educational level which were allowed to vary across time. The three educational categories were based on the highest level of education achieved, and were categorised as ‘higher education’, equivalent to bachelor level or higher; ‘intermediate education’, equivalent to upper secondary or short tertiary level; or ‘lower education’, equivalent to lower secondary or lower level. Living arrangements were dichotomised as ‘living with other individuals’ or ‘living alone’. Rates per 1000 person-years with SEs were estimated marginal means derived from the full-adjusted Poisson regression models.

For the within-individual associations, the follow-up time was split into periods of treatment and non-treatment. An individual was defined as exposed to treatment during the interval between two dispensed prescriptions unless prescription were issued more than 3 months apart.^20^ This is consistent with prescribing practices for these medications. The start of treatment was defined as the date of the first prescription, and the end of treatment was defined as the date after 3 months of the last prescription. We censored observations at the end of follow-up, or in the event of death or emigration. We calculated incidence rate ratios using conditional Poisson regression. In this self-controlled case series (SCCS) analysis, which is a case-only method, the relative risk is based on within-individual comparisons rather than between-individual comparisons, with each individual contributing to both exposed and unexposed observation time.^24^ The model requires thus that each individual has both at least one exposed and unexposed period. This approach provides implicit adjustment for all observed and unobserved time-invariant characteristics of individuals, such as gender and genetic disposition, and it also allows control for observed time-varying covariates, such as age.^24^ Therefore, the estimated coefficients of these models are not biased because of omitted time-invariant characteristics. Also, we performed additional within-individual analyses in which we included individuals who had only dispensed prescriptions of lithium (n=993), and those who were dispensed both lithium and antipsychotics (n=6744). The within-individual analyses were adjusted for age and co-occurring use of antidepressant and hypnotics/anxiolytics. This SCCS method was also used for the negative control analyses, sensitivity analyses and analyses for specific antipsychotic medication separately, in which we only included individuals who had a dispensed prescription for the single medication in question. A p value less than 0.05 was deemed statistically significant. All analyses were conducted using Stata software, V.16.1 (Stata Corp., College Station, Texas, USA).

### Sensitivity analyses

To corroborate our results, we explored to what extent reverse causation bias may have contributed by excluding crime events or suicide attempts that had occurred between 7 and 30 days prior to the start of antipsychotic prescription. We also used the SCCS method for the sensitivity analyses.

## Findings

Among 166 328 patients with diagnosed personality disorder, 79 253 (48%) had at least one dispensed antipsychotic between 1 January 2007 and 31 December 2016. Table 1 shows the baseline characteristics of these patients. Those who had dispensed prescriptions were more often men, less educated, more likely to be living alone and to be dispensed other psychotropic medications. The four most common personality disorder subcategories were emotionally unstable (code F60.3), unspecified (code F60.9), anxious (avoidant) (code F60.6) and mixed and other (code F61). Co-occurring categories of personality disorder was common.

**Table 1:** Characteristics of persons with diagnosed personality disorder dispensed antipsychotic medication and those not dispensed medication in 2007–2016 (n=166 328)

	No medication cohort	Medication cohort	P value
	Person-years (%)	Person-years (%)	
Age group, years			P<0.001
18–24	125 122 (17)	102 560 (15)	
25–34	183 091 (26)	165 916 (24)	
35–49	247 802 (35)	258 785 (37)	
50–64	159 421 (22)	170 287 (24)	
Gender			P<0.001
Men	286 592 (40)	315 588 (45)	
Women	428 844 (60)	383 960 (55)	
Educational level			P<0.001
Higher	415 451 (58)	328 750 (47)	
Basic	299 985 (42)	368 798 (53)	
Living arrangement			P<0.001
Married or cohabiting	326 756 (46)	259 576 (37)	
Living alone	388 680 (54)	437 972 (63)	
Personality disorder*			P<0.001
Paranoid	2045 (2)	3365 (4)	
Schizoid	2762 (3)	3929 (5)	
Dissocial	3951 (5)	6793 (9)	
Emotionally unstable	34 249 (39)	42 987 (54)	
Histrionic	1554 (2)	2156 (3)	
Anankastic	2144 (3)	2373 (3)	
Anxious	13 179 (15)	12 859 (16)	
Dependent	4667 (5)	5645 (7)	
Other specific	4659 (5)	5420 (7)	
Unspecified	33 280 (38)	36 883 (47)	
Mixed and other	11 374 (13)	14 804 (19)	
Medication†			
Antipsychotics		79 253 (100)	
Antidepressants	49 325 (57)	63 765 (80)	P<0.001
Hypnotics/anxiolytics	35 638 (41)	56 494 (71)	P<0.001
Adrenergic inhalants	6934 (8)	8819 (11)	P<0.001
Total			
Individuals	87 075 (100)	79 253 (100)	
Person-years at risk	715 436 (100)	697 548 (100)	

*Number with proportion (%) in personality disorder refers to number of individuals that have been registered with the diagnosis in question during the follow-up.

†Number with proportion (%) in medication refers to number of individuals that have at least one dispense of antipsychotic medication during follow-up.

In between-individual analyses, compared with men who had not been dispensed medication for antipsychotics, men with dispensed antipsychotics had an adjusted incident rate ratio (IRR) of 1.95 (95% CI 1.84 to 2.06) for violent crime suspicions and 1.29 (95% CI 1.26 to 1.33) for suicidal behaviour (table 2). Corresponding IRRs among women were 3.11 (95% CI 2.80 to 3.47) for violent crime and 1.55 (95% CI 1.51 to 1.58) for suicidal behaviour. Compared with women, men had between 6-fold and 10-fold higher rates for violent criminality, whereas the rates were similar for suicidal behaviour.

**Table 2:** Association between antipsychotic medication and violent crime suspicions or suicidal behaviour in personality disorders as reported by incident rate ratios (IRR) derived from between-individual analyses

	Men				Women			
	Cases	Rate (SE)	IRR	95% CI	Cases	Rate (SE)	IRR	95% CI
Violent crime suspicions								
No medication (ref)	7116	26.9 (0.6)	1.00		1067	2.7 (0.1)	1.00	
Medication	17 166	52.4 (1.0)	1.95	1.84 to 2.06	3271	8.4 (0.3)	3.11	2.80 to 3.47
Suicidal behaviour								
No medication (ref)	26 901	112.6 (1.1)	1.00		35 788	97.9 (0.8)	1.00	
Medication	45 694	145.4 (1.2)	1.29	1.26 to 1.33	57 023	151.4 (1.1)	1.55	1.51 to 1.58

Medication refers to individuals that have at least one purchase of antipsychotic medication during the follow-up, while those in no medication group do not have any purchases of antipsychotics. Models are adjusted for age, education, living arrangement and use of antidepressants and hypnotics/anxiolytics.

To account for confounders that were constant within each patient during follow-up, we conducted within-individual analyses to compare rates of violent crime and suicidal behaviour in the same individual when they were both on and off medication (table 3). These models were adjusted for age and concomitant use of antidepressant and hypnotics/anxiolytics. We noted substantially lower rates of violent crime suspicions and suicidal behaviour both in men and women during periods of antipsychotic prescription. IRRs for violent criminality in men and women were 0.60 (95% CI 0.55 to 0.63) and 0.90 (95% CI 0.79 to 1.01), respectively. Men and women had risk ratios of the same magnitude for suicidal attempts with IRRs of 0.68 (95% CI 0.66 to 0.71) and 0.68 (95% CI 0.65 to 0.70), respectively. We also performed within-individual analyses for the association between specific antipsychotic medication and outcomes, where both men and women were included in the same models (online supplemental eTable 1). The observed associations, adjusted for age and co-occurring use of antidepressant and hypnotics/anxiolytics, were similar for the most common individual antipsychotics (risperidone, quetiapine and olanzapine).

10.1136/ebmental-2022-300493.supp1 Supplementary data

**Table 3:** Association between antipsychotic medication and violent crime suspicions or suicidal behaviour in personality disorders as reported by incident rate ratios (IRR) derived from within-individual analyses

	Men			Women		
	Cases	IRR	95% CI	Cases	IRR	95% CI
Violent crime suspicions						
No medication (ref)	6656	1.00		1241	1.00	
Medication	2309	0.60	0.55 to 0.63	813	0.90	0.79 to 1.01
Suicidal behaviour						
No medication (ref)	18 884	1.00		21 295	1.00	
Medication	10 227	0.68	0.66 to 0.71	11 886	0.68	0.65 to 0.70

Models are adjusted for age and use of antidepressants and hypnotics/anxiolytics.

**Figure 1:**
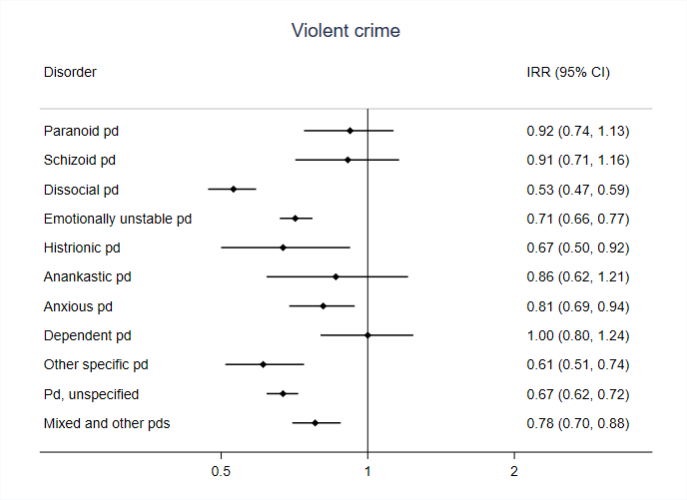
Incident rate ratios (IRR) derived from within-individual analyses for the association between antipsychotic medication and violent crime suspicions in specific personality disorders (PDs). Models are adjusted for age and use of antidepressants and hypnotics/anxiolytics.

**Figure 2:**
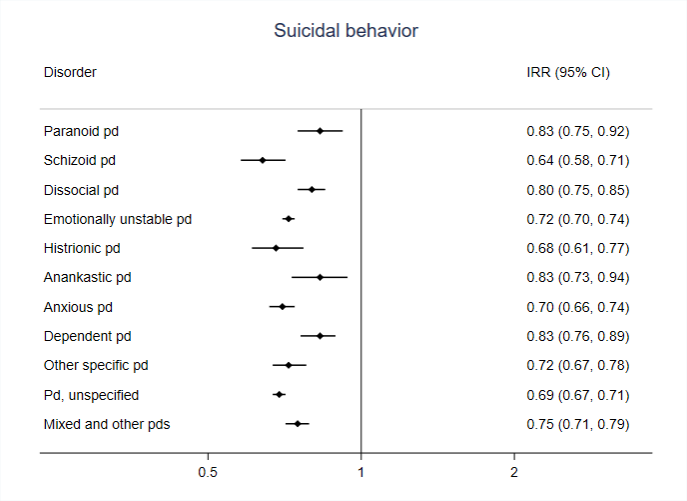
Incident rate ratios (IRR) derived from within-individual analyses for the association between antipsychotic medication and suicidal behaviour in specific personality disorders (PDs). Models are adjusted for age and use of antidepressants and hypnotics/anxiolytics.

We also conducted within-individual analyses separately for individuals diagnosed with specific personality disorders combining the sexes (figures 1 and 2). For violent crimes, the associations with medication were strongest for people with dissocial personality disorder with an IRR of 0.53 (95% CI 0.47 to 0.59), and clear associations were also observed for other specific personality disorders, including emotionally unstable (figure 1). For suicidal behaviour, associations with medication were large in all personality disorder subcategories (figure 2).

We conducted within-individual analyses including individuals who had only dispensed lithium, but this analysis was limited by relatively few individuals. When we examined those who had dispensed both antipsychotics and lithium, we found similar pattern of association but with less strong effects in men (online supplemental eTable 2). We performed within-individual negative control analyses to examine the association between adrenergic inhalants and adverse outcomes (table 4), and did not observe any significant associations between them and measured adverse outcomes in men or women.

**Table 4:** Incident rate ratios (IRR) derived from within-individual negative control analysis for the association between adrenergic inhalators and violent crime suspicions or suicidal behaviour in personality disorders

	Men			Women		
	Cases	IRR	95% CI	Cases	IRR	95% CI
Violent crime suspicions						
No medication (ref)	279	1.00		172	1.00	
Medication	168	1.02	0.81 to 1.30	87	0.87	0.64 to 1.18
Suicidal behaviour						
No medication (ref)	978	1.00		1935	1.00	
Medication	935	1.03	0.90 to 1.18	1785	0.96	0.87 to 1.06

Models are adjusted for age.

### Sensitivity analyses

To account for the potential bias induced by reverse causation (eg, a criminal offence or suicide attempt leading to a new antipsychotic prescription), we excluded violent crime suspicions and suicidal behaviour that had occurred within 7 up to 30 days before every medication period. The same patterns of association were found (online supplemental eTable 3).

## Discussion

This nationwide longitudinal study over 10 years of 166 328 patients with diagnosed personality disorder included 11 510 violent crime outcomes and 67 839 healthcare-presenting episodes of suicidal behaviour. Compared with periods when individuals were not on medication, antipsychotics were associated with 40% decrease in suspicions for violent crimes in men and 10% in women. Antipsychotic medication was also associated with lower rates of suicidal behaviour, which was reduced by 32% both in men and women during periods on antipsychotic medication. In subgroup analyses, the magnitude of the association varied across specific personality disorders for violent criminality but less for suicidal behaviour, with larger effects in dissocial personality disorder. In our negative control analysis, no significant associations were found between adrenergic inhalants, a commonly used medication class with negligible psychotropic effects, and adverse outcomes.

To our knowledge, this the first study investigating the association between antipsychotics treatment and the risk of violent crime or suicidal behaviour in people with diagnosed personality disorder. Evidence in other populations is consistent with our findings. In a general population study, violent crime fell by 45% in people receiving antipsychotics.^17^ In released prisoners, rates of violent reoffending were 42% lower during periods when individuals were dispensed antipsychotics in a within-individual study.^20^ In schizophrenia, a study found that clozapine use was associated with a 35% lower risk of suicidal behaviour.^21^

Our findings support the short-term use of antipsychotic medication for people with personality disorder. However, the absence of high-quality evidence on their clinical effectiveness and the elevated risk of metabolic and other adverse events associated with these medications are important reasons to be cautious about their longer-term use.^25^ Given underlying elevated levels of cardiovascular disease in personality disorder,^26^ the same level of enhanced physical health monitoring for those prescribed these drugs is necessary as people for individuals prescribed them for other reasons.^27 28^ We also found that adding lithium to antipsychotics did not materially change risk of outcomes investigated, and there were weaker associations in men. This would suggest that co-administration of lithium, which can exacerbate metabolic side effects, should not be routinely considered as a first-line strategy to prevent violence and suicidality.

Some possible mechanisms may explain our findings. Antipsychotics have effects on impulsivity directly,^29^ in addition to effects on hallucinations and delusions,^30^ which mediate the link between mental disorders and adverse outcomes. These medications may also reduce comorbid substance use,^31^ which increases risk. Moreover, antipsychotics may reduce emotional lability.^32^ It is also possible that non-specific factors, such as the instillation of hope or higher levels of contact with mental health services that people tend receive when prescribed these medications, may contribute to the reported associations.

While the association between antipsychotics and suicidal behaviour was consistently strong across individual personality disorders, there was more variation between individual personality disorders for the violent crime outcome. The strongest associations with antipsychotic prescriptions were in people with cluster B personality disorders (that include dissocial and emotionally unstable personality disorders). Cluster B disorders are linked with certain symptoms that mediate violent behaviour, such as impulsivity and emotional instability, which are one possible mechanism for the effects of antipsychotics in this population.

Strengths of our study include the use of nationwide high-quality registries with low rates of attrition during the follow-up. Some limitations need to be considered. First, although national registers on dispensed prescriptions are objective measures and are collected routinely, they do not provide information on whether medications were actually taken. The degree of non-adherence to dispensed medications may have led to overestimation or underestimation of the effect of treatment depending on whether outcomes occur or not during this period of misclassification. Second, our findings might be partially explained by reverse causation, in which violent crimes or suicide attempts increase the likelihood of the patients being prescribed antipsychotics. However, we did not observe evidence of such biases in our sensitivity tests. Third, there was a different direction of some associations when comparing the between and within-individual designs. This is likely due to the within-individual design allowing for adjustment of residual confounding. However, the within-individual approach does not allow for identification of the specific confounds that could explain the difference, and whether the confounds are more associated with the exposure (ie, antipsychotic prescription) or the outcome. Fourth, in our analyses on effects of specific antipsychotics, we could not perform separate analyses on violent crime for clozapine, a potentially effective drug in adverse outcome reduction, due to few prescriptions as a single antipsychotic. Fifth, comorbidity is common in personality disorders,^2^ and it might influence results. However, in the within-individual method we used, observed and unobserved time-invariant confounders, including certain comorbidities, were implicitly controlled for. For some other comorbidities, such as substance misuse, the design is limited, and findings need triangulation with other designs. A further limitation is that our findings may not generalise to individuals with personality disorders who have not been prescribed antipsychotic medication. This broader population may have less severe symptoms and behavioural problems, and therefore not potentially benefit from any medication effects.

In terms of the generalisability of the findings, although the epidemiology of personality disorders is poorly described,^2^ recent meta-analyses report a prevalence rate of 12%,^1^ and 10% in high-income countries.^33^ In Denmark, a recent study found that 11% of the community sample fulfilled the estimated screening criteria for a personality disorder.^34^ Prevalence of antipsychotic use is 1.7% in Scandinavia (including Denmark)^35^ and similar in the US population.^36^

In conclusion, we found that treatment with antipsychotics was associated with reduced risks for violent criminality and suicidal behaviour among individuals with personality disorder. This real-world study suggests that the potential effects of antipsychotics on suicidal behaviour and violence should be taken into account when considering treatment options for people with personality disorders.

## Data Availability

Data may be obtained from a third party and are not publicly available.

## References

[1] Volkert J , Gablonski T-C , Rabung S . Prevalence of personality disorders in the general adult population in Western countries: systematic review and meta-analysis. Br J Psychiatry 2018;213:709–15. 10.1192/bjp.2018.202 30261937

[2] Tyrer P , Reed GM , Crawford MJ , Classification CMJ . Classification, assessment, prevalence, and effect of personality disorder. Lancet 2015;385:717–26. 10.1016/S0140-6736(14)61995-4 25706217

[3] Fok ML-Y , Hayes RD , Chang C-K , et al . Life expectancy at birth and all-cause mortality among people with personality disorder. J Psychosom Res 2012;73:104–7. 10.1016/j.jpsychores.2012.05.001 22789412

[4] Soeteman DI , Hakkaart-van Roijen L , Verheul R , et al . The economic burden of personality disorders in mental health care. J Clin Psychiatry 2008;69:259–65. 10.4088/JCP.v69n0212 18363454

[5] Yu R , Geddes JR , Fazel S . Personality disorders, violence, and antisocial behavior: a systematic review and meta-regression analysis. J Pers Disord 2012;26:775–92. 10.1521/pedi.2012.26.5.775 23013345

[6] Olajide K , Tyrer P , Singh SP , et al . Likelihood and predictors of detention in patients with personality disorder compared with other mental disorders: a retrospective, quantitative study of mental health act assessments. Personal Ment Health 2016;10:191–204. 10.1002/pmh.1332 26992030

[7] Fazel S , Runeson B . Suicide. N Engl J Med 2020;382:266–74. 10.1056/NEJMra1902944 31940700 PMC7116087

[8] Yen S , Peters JR , Nishar S , et al . Association of borderline personality disorder criteria with suicide attempts: findings from the Collaborative longitudinal study of personality disorders over 10 years of follow-up. JAMA Psychiatry 2021;78:187–94. 10.1001/jamapsychiatry.2020.3598 33206138 PMC7675214

[9] Li Z , Page A , Martin G , et al . Attributable risk of psychiatric and socio-economic factors for suicide from individual-level, population-based studies: a systematic review. Soc Sci Med 2011;72:608–16. 10.1016/j.socscimed.2010.11.008 21211874

[10] Bateman AW , Gunderson J , Mulder R . Treatment of personality disorder. Lancet 2015;385:735–43. 10.1016/S0140-6736(14)61394-5 25706219

[11] Storebø OJ , Stoffers-Winterling JM , Völlm BA , et al . Psychological therapies for people with borderline personality disorder. Cochrane Database Syst Rev 2020;5:CD012955. 10.1002/14651858.CD012955.pub2 32368793 PMC7199382

[12] Stoffers-Winterling J , Storebø OJ , Lieb K . Pharmacotherapy for borderline personality disorder: an update of published, unpublished and ongoing studies. Curr Psychiatry Rep 2020;22:37. 10.1007/s11920-020-01164-1 32504127 PMC7275094

[13] Bohus M , Stoffers-Winterling J , Sharp C , et al . Borderline personality disorder. Lancet 2021;398:1528–40. 10.1016/S0140-6736(21)00476-1 34688371

[14] Paton C , Crawford MJ , Bhatti SF , et al . The use of psychotropic medication in patients with emotionally unstable personality disorder under the care of UK mental health services. J Clin Psychiatry 2015;76:e512–8. 10.4088/JCP.14m09228 25919844

[15] Crawford MJ , Kakad S , Rendel C , et al . Medication prescribed to people with personality disorder: the influence of patient factors and treatment setting. Acta Psychiatr Scand 2011;124:396–402. 10.1111/j.1600-0447.2011.01728.x 21707555

[16] Cristea IA , Gentili C , Cotet CD , et al . Efficacy of psychotherapies for borderline personality disorder: a systematic review and meta-analysis. JAMA Psychiatry 2017;74:319–28. 10.1001/jamapsychiatry.2016.4287 28249086

[17] Fazel S , Zetterqvist J , Larsson H , et al . Antipsychotics, mood stabilisers, and risk of violent crime. Lancet 2014;384:1206–14. 10.1016/S0140-6736(14)60379-2 24816046 PMC4165625

[18] Sariaslan A , Leucht S , Zetterqvist J , et al . Associations between individual antipsychotics and the risk of arrests and convictions of violent and other crime: a nationwide within-individual study of 74 925 persons. Psychol Med 2021:1–9. Online ahead of print.. 10.1017/S0033291721000556 PMC9811342 33691828

[19] Bhavsar V , Kosidou K , Widman L , et al . Clozapine treatment and offending: a within-subject study of patients with psychotic disorders in Sweden. Schizophr Bull 2020;46:303–10. 10.1093/schbul/sbz055 31150553 PMC7442333

[20] Chang Z , Lichtenstein P , Långström N , et al . Association between prescription of major psychotropic medications and violent Reoffending after prison release. JAMA 2016;316:1798–807. 10.1001/jama.2016.15380 27802545 PMC5100822

[21] Taipale H , Lähteenvuo M , Tanskanen A , et al . Comparative effectiveness of antipsychotics for risk of attempted or completed suicide among persons with schizophrenia. Schizophr Bull 2021;47:23–30. 10.1093/schbul/sbaa111 33428766 PMC7824993

[22] Gasse C , Danielsen AA , Pedersen MG , et al . Positive predictive value of a register-based algorithm using the Danish national registries to identify suicidal events. Pharmacoepidemiol Drug Saf 2018;27:1131–8. 10.1002/pds.4433 29664233

[23] Mors O , Perto GP , Mortensen PB . The Danish psychiatric central research register. Scand J Public Health 2011;39:54–7. 10.1177/1403494810395825 21775352

[24] Whitaker HJ , Farrington CP , Spiessens B , et al . Tutorial in biostatistics: the self-controlled case series method. Stat Med 2006;25:1768–97. 10.1002/sim.2302 16220518

[25] Kendall T , Pilling S , Tyrer P , et al . Borderline and antisocial personality disorders: summary of NICE guidance. BMJ 2009;338:b93. 10.1136/bmj.b93 19176682

[26] Hall K , Barnicot K , Crawford M , et al . A systematic review of interventions aimed at improving the cardiovascular health of people diagnosed with personality disorders. Soc Psychiatry Psychiatr Epidemiol 2019;54:897–904. 10.1007/s00127-019-01705-x 30929043

[27] Cooper SJ , Reynolds GP , et al, With expert co-authors (in alphabetical order): . BAP guidelines on the management of weight gain, metabolic disturbances and cardiovascular risk associated with psychosis and antipsychotic drug treatment. J Psychopharmacol 2016;30:717–48. 10.1177/0269881116645254 27147592

[28] Paton C , Crawford MJ , Bhatti SF , et al . The use of psychotropic medication in patients with emotionally unstable personality disorder under the care of UK mental health services. J Clin Psychiatry 2015;76:e512–8. 10.4088/JCP.14m09228 25919844

[29] van Schalkwyk GI , Beyer C , Johnson J , et al . Antipsychotics for aggression in adults: a meta-analysis. Prog Neuropsychopharmacol Biol Psychiatry 2018;81:452–8. 10.1016/j.pnpbp.2017.07.019 28754408

[30] Johnsen E , Sinkeviciute I , Løberg E-M , et al . Hallucinations in acutely admitted patients with psychosis, and effectiveness of risperidone, olanzapine, quetiapine, and ziprasidone: a pragmatic, randomized study. BMC Psychiatry 2013;13:241. 10.1186/1471-244X-13-241 24079855 PMC3850701

[31] Krause M , Huhn M , Schneider-Thoma J , et al . Efficacy, acceptability and tolerability of antipsychotics in patients with schizophrenia and comorbid substance use. A systematic review and meta-analysis. Eur Neuropsychopharmacol 2019;29:32–45. 10.1016/j.euroneuro.2018.11.1105 30472164

[32] Van den Eynde F , Senturk V , Naudts K , et al . Efficacy of quetiapine for impulsivity and affective symptoms in borderline personality disorder. J Clin Psychopharmacol 2008;28:147–55. 10.1097/JCP.0b013e318166c4bf 18344724

[33] Winsper C , Bilgin A , Thompson A , et al . The prevalence of personality disorders in the community: a global systematic review and meta-analysis. Br J Psychiatry 2020;216:69–78. 10.1192/bjp.2019.166 31298170

[34] Bach B , Kongerslev MT , Simonsen E . Prevalence and structure of self-other problems in SAPAS screening for personality disorder in a national sample. Personal Ment Health 2020;14:175–85. 10.1002/pmh.1470 31762203

[35] Højlund M , Pottegård A , Johnsen E , et al . Trends in utilization and dosing of antipsychotic drugs in Scandinavia: comparison of 2006 and 2016. Br J Clin Pharmacol 2019;85:1598–606. 10.1111/bcp.13945 30927284 PMC6595354

[36] Dennis JA , Gittner LS , Payne JD . Characteristics of U.S. adults taking prescription antipsychotic medications, National health and nutrition examination survey 2013-2018. BMC Psychiatry 2020;20:483. 33004022 10.1186/s12888-020-02895-4 PMC7528276

