# Supplementary material for: Associations between antipsychotics and risk of violent crimes and suicidal behaviour in personality disorder
Source: Evid Based Ment Health. 2022 Oct 25;25(e1):e58–64. doi: 10.1136/ebmental-2022-300493 (PMC9811101; doi:10.1136/ebmental-2022-300493)
Supplement: Supplementary data [file ebmental-2022-300493supp001.pdf]

eTable 1. Incident rate ratios (IRR) with 95% confidence intervals (95% CI) derived from within-individual analysis for the association between specific antipsychotic medication and violent crime among male and suicidal behavior both among men and women diagnosed with personality disorders.

	Violent crime			Suicidal behavior		
	Cases	IRR	95% CI	Cases	IRR	95% CI
Risperidone						
No medication (ref)	235	1.00		1315	1.00	
Medication	77	0.55	0.36, 0.84	599	0.66	0.56, 0.77
Olanzapine						
No medication (ref)	365	1.00		1528	1.00	
Medication	82	0.51	0.36, 0.72	764	0.67	0.59, 0.77
Quetiapine						
No medication (ref)	1419	1.00		9156	1.00	
Medication	156	0.50	0.41, 0.62	1710	0.55	0.51, 0.59
Clozapine						
No medication (ref)	16	-		38	1.00	
Medication	15	-	-	66	0.57	0.31, 1.08
Other antipsychotics						
No medication (ref)	1330	1.00		9876	1.00	
Medication	307	0.54	0.45, 0.64	4420	0.65	0.62, 0.69

Models are adjusted for age and use of antidepressants and hypnotics/anxiolytics. Only patients without other prescribed antipsychotics than in question included in analyses. The analysis for the association between clozapine and violent crime was not feasible due to a low number of cases.

eTable 2. Incident rate ratios (IRR) with 95% confidence intervals (95% CI) derived from within-individual analysis for the association between psychotropic medication and violent crime or suicidal behavior among individuals diagnosed with personality disorders.

	Men			Women		
	Cases	IRR	95% CI	Cases	IRR	95% CI
Violent crime						
No medication (ref)	184	1.00		58	1.00	
Medication	163	0.87	0.66, 1.16	86	0.78	0.49, 1.24
Suicidal behavior						
No medication (ref)	920	1.00		1,630	1.00	
Medication	1,066	0.86	0.76, 0.98	1,931	0.75	0.69, 0.83

Psychotropic medication refers to antipsychotics and lithium. Individuals in the analyses are those who have dispensed both antipsychotics and lithium. Models are adjusted for age and use of antidepressants and hypnotics/anxiolytics.

eTable 3. Incident rate ratios (IRR) with 95% confidence intervals (95% CI) derived from within-individual analysis for the association between antipsychotic medication and violent crime or suicidal behavior among individuals diagnosed with specific personality disorders.

	Violent crime			Suicidal behavior		
	Cases	IRR	95% CI	Cases	IRR	95% CI
Paranoid pd						
No medication (ref)	377	1.00		1602	1.00	
Medication	292	1.07	0.86, 1.33	1589	0.94	0.85, 1.05
Schizoid pd						
No medication (ref)	287	1.00		1715	1.00	
Medication	255	0.94	0.74, 1.20	1508	0.78	0.70, 0.86
Dissocial pd						
No medication (ref)	2053	1.00		4100	1.00	
Medication	758	0.58	0.52, 0.65	3224	0.90	0.85, 0.96
Emotionally unstable pd						
No medication (ref)	3829	1.00		21570	1.00	
Medication	2077	0.77	0.72, 0.83	14543	0.82	0.80, 0.85
Histrionic pd						
No medication (ref)	178	1.00		985	1.00	
Medication	146	0.72	0.52, 0.99	1068	0.83	0.73, 0.95
Anankastic pd						
No medication (ref)	173	1.00		1115	1.00	
Medication	122	0.74	0.56, 1.01	887	0.97	0.85, 1.11
Anxious pd						
No medication (ref)	764	1.00		5766	1.00	
Medication	539	0.84	0.72, 0.98	4203	0.83	0.78, 0.88
Dependent pd						
No medication (ref)	303	1.00		2445	1.00	
Medication	313	1.11	0.89, 1.39	2461	0.96	0.88, 1.04
Other specific pd						
No medication (ref)	560	1.00		2676	1.00	
Medication	331	0.70	0.57, 0.85	2331	0.85	0.78, 0.92
Pd, unspecified						
No medication (ref)	3817	1.00		19137	1.00	
Medication	1885	0.72	0.67, 0.78	12089	0.81	0.78, 0.84
Mixed and other pds						
No medication (ref)	1428	1.00		7413	1.00	
Medication	816	0.84	0.75, 0.95	5352	0.85	0.81, 0.90

Sensitivity test: Within-individual associations between prescriptions of antipsychotic medications and violent crime and suicidal behavior where crimes or suicide attempts occurring between 7 to 30 days before the antipsychotic exposure were excluded. Models are adjusted for age and use of antidepressants and hypnotics/anxiolytics.
